# Sensorimotor, Attentional, and Neuroanatomical Predictors of Upper Limb Motor Deficits and Rehabilitation Outcome after Stroke

**DOI:** 10.1155/2021/8845685

**Published:** 2021-04-01

**Authors:** Daniela D'Imperio, Zaira Romeo, Lorenza Maistrello, Eugenia Durgoni, Camilla Della Pietà, Michele De Filippo De Grazia, Francesca Meneghello, Andrea Turolla, Marco Zorzi

**Affiliations:** ^1^IRCCS San Camillo Hospital, Venice, Italy; ^2^Department of General Psychology and Padova Neuroscience Center, University of Padova, Italy

## Abstract

The rehabilitation of motor deficits following stroke relies on both sensorimotor and cognitive abilities, thereby involving large-scale brain networks. However, few studies have investigated the integration between motor and cognitive domains, as well as its neuroanatomical basis. In this retrospective study, upper limb motor responsiveness to technology-based rehabilitation was examined in a sample of 29 stroke patients (18 with right and 11 with left brain damage). Pretreatment sensorimotor and attentional abilities were found to influence motor recovery. Training responsiveness increased as a function of the severity of motor deficits, whereas spared attentional abilities, especially visuospatial attention, supported motor improvements. Neuroanatomical analysis of structural lesions and white matter disconnections showed that the poststroke motor performance was associated with putamen, insula, corticospinal tract, and frontoparietal connectivity. Motor rehabilitation outcome was mainly associated with the superior longitudinal fasciculus and partial involvement of the corpus callosum. The latter findings support the hypothesis that motor recovery engages large-scale brain networks that involve cognitive abilities and provides insight into stroke rehabilitation strategies.

## 1. Introduction

Stroke survivors may suffer from motor, cognitive, and/or psychological deficits, with conjoined consequences for the course of rehabilitation as well as for the quality of life. The presence of motor impairments (i.e., hemiparesis, coordination problems, and spasticity) is very common and it evidently affects patients' everyday autonomy, with a high variability of recovery that depends on both spontaneous and rehabilitation-induced gains [1].

The rehabilitation of neurological motor impairments is based on motor learning principles within complex sensorimotor and cognitive processes [2]. Repracticing the execution of goal-directed actions requires some planning and computational steps that engage connections among various brain areas [3, 4]. This hierarchical process goes from the sensory integration between bodily information learned from previous experiences [5] and on-line movements and context [4, 6] up to the execution of voluntary movements. On one side, the interpatients variability in preserved sensorimotor abilities is critical for functional motor skills [7], on-going control [8], and prognosis [9]. On the other side, the cognitive system supports motor execution, in terms of planning the computational steps and of attention on internal and external sensorimotor feedbacks to monitor and adjust the performance [6, 10, 11]. As a matter of fact, stroke patients with motor deficits mainly have difficulties to cope with everyday actions, which often involve high attentional load due to multitasking demands (e.g., walk and avoid obstacles), thereby worsening sensory inputs' processing [12] and motor execution [13]. Indeed, the major goal of motor rehabilitation is the recovery of everyday life abilities.

Recent innovative approaches for motor rehabilitation with technology-based (hereafter, TB) techniques aim to resemble the ecological environments, where behavior is demanding and cognitive abilities may be involved [2, 14]. TB methods are based on interactive action-feedback simulation software, which engages patients into real-world-like scenarios [2, 15, 16] and supports motor recovery, as demonstrated for upper limb rehabilitation [17–20]. Nevertheless, a recent Cochrane review noted that most studies of TB rehabilitation (i.e., using virtual reality) usually exclude patients with severe cognitive deficits, thereby prompting for further investigations on cognitive abilities as covariate in motor training outcome [21].

Considering the integration of motor and cognitive systems underlying motor learning [2], a crucial challenge is to exploit their functioning at a neural level in neurological patients. It is well known that lesions in primary and secondary motor cortices [2], corticospinal tract [22], and interhemispheric connections [23] affect the severity of upper limb impairments. However, recent results highlight the role of brain connectivity encompassing bilateral motor, premotor, and frontal areas [24] and forming a large-scale temporofrontoparietal functional network [25–28]. The neural plasticity of this large-scale network may give insight into the interpatients variability in motor recovery [29, 30] within the cooccurrence of cognitive deficits [31]. In particular, a clear link between motor and attentional abilities is shown by the neglect syndrome [32, 33], a visuospatial attention deficit in orienting and reporting relevant stimuli on the contralesional side of space [34], mainly occurring after right hemisphere stroke ([35, 36], but see [37]). More generally, the efficacy of motor rehabilitation may depend on many factors that include patients' residual abilities [1, 9], training approaches [15], and type of neuroanatomical impairments [3, 38].

The goal of the present retrospective study was to investigate how the sensorimotor and attention systems contribute to motor recovery of upper limb impairments following TB rehabilitation. We only considered patients who underwent a TB physiotherapy program in order to have a consistent rehabilitation approach, which was also closer to real-life requests. We examined the influence of selective attention skills in the whole sample of patients, whereas for a subgroup of right stroke patients we additionally examined the role of visuospatial orienting abilities. To complete the picture, we also inspected the neural structures associated with both initial and postrehabilitation motor performance. We examined the association with the structural lesion [39] as well as with the white-matter disconnections [40]. The latter represents a novel approach to examine direct structural disconnections after a focal lesion [40] and provides valuable knowledge about the mapping between connectivity and behavior [24].

## 2. Materials and Methods

### 2.1. Participants

Stroke patients hospitalized between 2010 and 2017 at IRCCS San Camillo Hospital (Venice, Italy) were considered for the retrospective study.

Patients were initially inspected for the following features: adult age, first stroke (from ischemic or hemorrhagic etiology), and availability of a brain structural MRI scan. Consequently, inclusion criteria were applied for (1) presence of unilateral brain lesion, (2) completion of upper limb TB rehabilitation protocol, and (3) administration of the attentive matrices test [41]. Additional exclusion criteria were implemented to take in consideration only patients who were likely to benefit from the motor rehabilitation: (1) presence of other neurological and psychiatric conditions in medical history assessed by available neurological tests and/or brain MRI scan (i.e., clinical signs of probable neurodegenerative deficits), (2) chronic stroke lesion (>1.5 years from onset), (3) pretreatment motor function of the upper limb showing negligible (values at the Upper Extremity Fugl-Meyer Assessment scale in the range 60-66, for potential ceiling effect) or very severe impairments (values in the range 0-6, for potential floor effect), which could impact the scale's sensitivity [42], and (4) long distance (>3 months) between assessment of attention and TB rehabilitation treatment.

**Figure 1:**
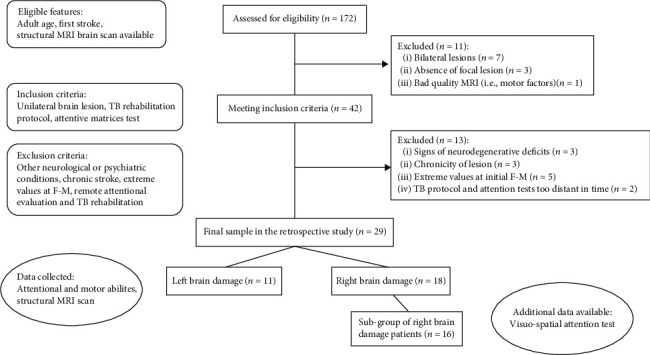
Enrollment flowchart. MRI: Magnetic Resonance Imaging; TB: Technology-based; F-M UE: Fugl-Meyer Upper Extremity test.

From the primary eligibility screening, 42 patients satisfied all the inclusion criteria, but other 13 patients were ruled out for exclusion conditions. The final sample consisted of 29 patients (mean age = 62.41 ± 11.87 years, mean education = 11.41 ± 4.50 years, mean time from onset = 7.18 ± 4.60 months), 11 with left (LBD) and 18 with right brain damage (RBD) (see Figure 1 for study inclusion flowchart; complete patients' data are provided in Supplementary materials). The study adhered to the Declaration of Helsinki and to the Italian regulation (Legislative Decree n. 211/2003; Ministry Decree 17 December 2004) for experimental studies in health care. The Ethical Committee for Clinical Research of the IRCCS San Camillo Hospital approved two studies to enroll patients after informed consent (Prot. 2013.11, registration at ClinicalTrials.gov NCT02234531 with virtual reality, and Prot. 2014.14 – sERF, registration at ClinicalTrials.gov NCT03207490 with AMADEO).

### 2.2. Cognitive Data

All patients underwent a neuropsychological assessment, but not consistently for the whole sample due to the retrospective design of the study. For a description of the sample, we recorded the tests present for at least 50% of the patients. These tests explored the following: general cognitive abilities (Minimental scale examination—MMSE [43]), reasoning (Raven's progressive matrices; [44]), short-term memory (Forward digit span, Spinnler and Tognoni, 1987), long-term memory (Rey figure - delayed; Caffarra et al., 2002), working memory (Backward digit span, [41]), and constructive apraxia with simple and complex figures (Copy of drawing; Spinnler and Tognoni, 1987; Rey figure - copy; [45]).

For the purpose of exploring attentional influences on motor rehabilitation responsiveness, we collected attentional test data. Selective attention was evaluated by the attentional matrices test, which is suitable for examining both RBD and LBD stroke patients [41]. In this test, patients are required to cross out some target numbers (1, 2, or 3) in three different numerical matrices within 45 seconds (overall range 0-60). Additionally, the assessment of visuospatial attention through the Behavioral Inattention Test (BIT) [46] was available for almost all of the right brain-damaged patients (16 out of 18 patients). The BIT includes 6 subtests (cancellation of lines, letters or stars, line bisection, figure copy, and drawing) to evaluate difficulties in visuospatial attention, and it is routinely used to assess the presence of neglect. BIT subtests highlight slightly different types of neglect, but only the cancellation tasks directly require visual scanning in the peripersonal space [47]. In particular, the Stars cancellation subtest requires to mark the little stars (range 0-54) in a page of confounders (big stars and words), thereby complicating visual scanning performance to yield a sensitive evaluation of neglect. Performance in the BIT Stars test was therefore used for the statistical analyses.

### 2.3. Motor Data

All the patients completed a physiotherapy rehabilitative program, which consisted of two different trainings: a traditional rehabilitation (TR) treatment and an additional one with TB technologies. Each treatment lasted for 1 hour/day for 5 sessions (3 weeks), 30 h overall. Both trainings were tailored to the patient's motor residual capabilities with progressive exercises' targets. Their combined responsiveness (TR+TB) was tested by the Upper Extremity section of the Fugl-Meyer Assessment scale.

Specifically, the TR exercises targeted the whole body to improve the patient's autonomy. The specific exercises for the upper limb consisted of passive, assisted, and active mobilizations in all free directions [48], driven by the physiotherapist. The TB rehabilitation protocol focused only on the upper limb's exercises in an ecological virtual setting, with the support of technologies that provided on-line reinforcement feedbacks. The protocol could use virtual reality software or the AMADEO robot, which are specifically applied for the rehabilitation of the upper limb with slight differences for the trained muscular districts. The exercises with virtual reality focus on the elbow and shoulder/proximal upper limb, with a 3D motion-tracking system (Polhemus 3Space FasTrak, Vermont, U.S.A.) as described by Piron and colleagues [2]. The AMADEO robot (Tyromotion GmbH Graz, Austria) treatment is based on detection and control of fingers' flexors and extensors through surface electromyography signals [19]. The choice of TB protocol was driven by clinical judgment and in particular by the residual abilities of the individual patient.

For the evaluation of sensorimotor abilities, all patients underwent a complete clinical assessment before treatment by (i) Modified Ashworth scale [49], for spasticity of five upper limb's muscle (total value was computed as the sum of each muscle, ranging from 0 to 20 as increasing severity), and (ii) Reaching Performance scale (range 0-36) ([50], for the upper limb reaching abilities. Additionally, the use of the Fugl-Meyer (F-M) scale [42] was considered separately for (iii) Sensation (range 0-24), rating impairment of tactile and proprioception sensation; (iv) Joints amplitude (range 0-48) rating range of motion and pain associated with passive mobilization of the upper limb; and (v) Upper Extremity (UE) (range 0-66) for overall assessment of upper limb motor function. The F-M UE subscale was readministered after rehabilitation as the primary measure to register possible changes between pre- and post-treatment performance [51].

### 2.4. Brain Lesion and Disconnection Preprocessing

All patients had a T1-weighted image from a 1.5 T Philips MRI scanner. As a first step, automated brain lesions segmentation was obtained using the Lesion Identification with Neighborhood Data Analysis software (LINDA [52]). The resulting lesion mask (in native MRI space) was visually inspected and manually corrected with ITK-snap software [53] by two researchers (RZ and DE) and the supervision of a neurologist in the case of slight differences between LINDA results and original T1 scans. Finally, to allow comparisons across patients, the lesion was spatially registered to a standard template using the pipeline of the BCBtoolkit software (http://toolkit.bcblab.com/) [40] (also see [24]). The individual lesion was replaced with healthy tissue of the contralateral hemisphere in an enantiomorphic method [54] to allow MRI scans and lesion masks' normalization to a MNI152 space (with 2∗2∗2 millimeters voxel size) with diffeomorphic deformation [55]. A quality check on the registration step was carried out through visual inspection.

The probable lesioned tracts were extracted using the BCBtoolkit Disconnectome maps tool [40]. In this approach, the individual lesion map was used as a seed for the tractography in TrackVis (http://trackvis.org/), by taking into account the interindividual variability from a healthy controls' dataset (as in [56]). In the resulting disconnections maps, voxels represent only disconnected tracts above the conventional probability threshold of 50% [40]. Note that values in the maps correspond to the maximum lesioned-streamline localization probabilities, not disconnection probabilities.

## 3. Data Analysis

### 3.1. Behavioral Data Analysis

In order to control descriptive difference across the sample, a first direct comparison between patients was run in relation to the side of lesion (LBD vs. RBD) on available neuropsychological assessment and experimental data (i.e., demographic, neurological, motor, and attentional), by means of T-test or Wilcoxon Test for continuous and ordinal data or Chi^2^ test for frequencies.

The inspection of motor rehabilitation responsiveness was run on the F-M UE outcome. Previous studies have shown that the initial severity of deficit is predictive of the behavioral recovery [57, 58]. Therefore, we computed a “F-M UE recovery index” [((posttreatment F − M UE − pretreatment F − M UE)/pretreatment F − M UE)∗100] to detect motor changes weighted by the pretreatment residual performance [57, 58]. Note that a raw measure of change (i.e., post–pre) does not consider the patient's initial ability and it would miss its impact on the performance gains. After controlling that its distribution did not diverge from normality using the Shapiro-Wilk test, this index was used as a dependent variable to analyze the association of motor improvement to all other collected data by means of a linear regression model. As in previous studies with a similar goal [59], a forward stepwise approach permits to sequentially introduce the variables in accordance with correlations to the dependent variable (the full correlation matrix is reported in Tables 5S-6S in Supplementary materials). The model fit was assessed by log-likelihood tests to compare models' residuals by Chi^2^ tests (entering those with *p* < 0.10), including all those factors that help explaining variance, but do not prevent model convergence [60]. Moreover, the robustness of the stepwise regression results was assessed using an alternative method, the best subset regression [61]. The latter generates models from all possible predictors' combinations, which are then compared in terms of goodness-of-fit. These results are reported in supplementary materials. Notably, the most conservative model contained the same predictors as the stepwise regression.

In the model, associations to the F-M UE recovery index were computed for the following independent variables: demographic information (i.e., age, gender, and education), clinical parameters (i.e., etiology, time from onset, damaged hemisphere, lesion volume, and type of TB motor training), attentional deficits (values at the attentional matrices test), and pretreatment upper limb residual motor performance. For the latter, a dimensionality reduction was carried out using principal component analysis (PCA) with oblique rotation on all collected pretreatment motor tests (i.e., Modified Ashworth, Reaching test, and all F-M subscales). Following Corbetta and colleagues (62; also see [24, 57]), we used the first principal component as “motor factor” score in all subsequent analyses, as it accounted for most of the variance (>60%, see Supplementary materials). The motor factor score is therefore highly representative of the motor tests and its use for regression modeling prevents the problem of including several correlated tests as predictors.

We also carried out an exploratory analysis to investigate the role of visuospatial attention in a subgroup of RBD patients for whom the BIT Stars score was available (16 out of 18). This test evaluates the visuospatial orienting component of attention, which is more frequently impaired following right hemisphere stroke ([36], but see [37]) and might be a better predictor of motor recovery compared to the more general index of selective attention available for the whole sample. The BIT Stars score was entered as an additional predictor variable in the regression analysis.

Analyses were run using the software R (R Core Team, 2018), using the package car [62].

### 3.2. Neuroimaging Data Analysis

To overcome the problem of small sample size, the lesion data were aligned onto a single hemisphere by flipping left lesion masks and disconnection maps into the space of the right hemisphere.

Firstly, an overlay map was created separately for lesions and disconnections. These maps allow us to depict the most overlapped damaged areas and to describe their localization. Afterwards, statistical analyses were separately run for lesions masks and disconnection maps, with a voxel-based lesion mapping (VLSM) method [39, 63] using the NPM program in the MRIcron software (http://www.cabiatl.com/mricro/mricron/index.html). The VLSM approach permits to explore strong lesion-deficit associations within a small neurological sample [64], by independently comparing all damaged voxels in a mass-univariate design [65, 66].

Two separate VLSM analyses were computed to estimate damaged voxels that predict the lower values at pretreatment F-M UE and at F-M UE recovery index, both for grey matter lesions and white matter disconnections. For instance, VLSM results report the damaged areas associated with residual abilities and motor recovery, respectively. Analyses were run using nonparametric Brunner-Menzel (BM) analysis on each voxel within the lesion mask for continuous behavioral data [67], controlling for lesion volumes as covariate, with voxel-level false discovery rate correction for multiple comparisons. With an atlas-based approach for identification [68], VLSM results were overlapped to the probabilistic Harvard-Oxford atlas [69] and the human brain atlas for single tracts [70] to label and identify the damaged voxels in grey structures and white matter tracts (see Supplementary materials for details).

## 4. Results

### 4.1. Descriptive Information

In order to ensure comparable groups, all relevant behavioral data were compared between LBD and RBD patients. RBD patients reported lower motor abilities in pre- and post-treatment assessments, but not in the F-M UE recovery index (Table 1). Other neuropsychological data was not available for the whole sample but is reported in the supplementary materials for descriptive purpose (Table 3S).

### 4.2. Motor Rehabilitation Responsiveness

The F-M UE recovery index did not diverge from a normal distribution (Shapiro-Wilk test, *W* = 0.949, *p* = 0.102), which is appropriate for linear regression modeling. In the resulting model, the F-M UE recovery index was predicted by age (*p* < 0.001), motor factor (*p* < 0.001), affected hemisphere (*p* = 0.009), and attentive matrices (*p* = 0.047) (see Table 2). No significant relation to other independent variables was found. The model yielded a very good fit, with *R*^2^ = 0.661 (adjusted *R*^2^ = 0.587, F − statistic′s Test = 8.969, *p* < 0.001). The residuals of the model did not diverge from a normal distribution (*W* = 0.957, *p* = 0.273).

An additional regression analysis on pretreatment F-M UE scores, reported in supplementary materials, revealed that initial motor performance was only influenced by time from stroke onset.

The F-M UE recovery index for the subgroup of right hemisphere stroke patients was still not statistically different from a normal distribution (*W* = 0.894, *p* = 0.065). Regression modeling showed that the recovery index was predicted by age (*p* < 0.001), time from onset (*p* = 0.034), and BIT Stars test (*p* = 0.033) (Table 3). No other predictor was significant. The model yielded *R*^2^ = 0.713 (adjusted *R*^2^ = 0.641, F − statistic′s Test = 9.936, *p* = 0.001), and the residuals did not diverge from a normal distribution (*W* = 0.944, *p* = 0.399).

### 4.3. Lesion and Disconnection Data

**Figure 2:**
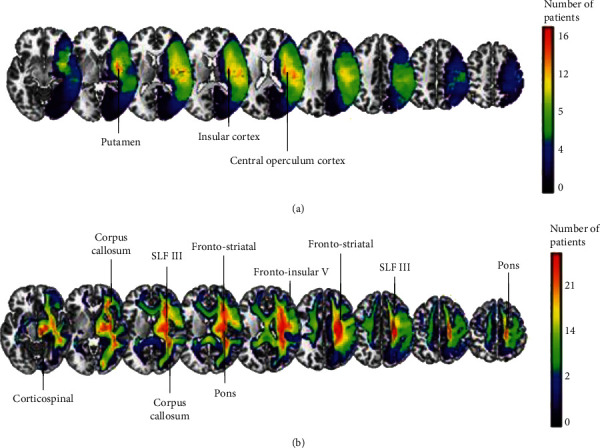
Overlay maps of lesions (a) and white-matter disconnections (b) on a standard brain MNI template. The color scale represents the number of patients.

Maximum lesion overlap was found in 17 patients (58.62%), and it mainly involved putamen, insular, temporal, and central operculum cortices (Figure 2(a)).

In terms of disconnected tracts, maximum overlap was found in 25 patients (86.21%). The most damaged tracts in percentage across all patients were corticospinal tract, corpus callosum, corticopontine, frontostriatal, frontoinsular tract V, and superior longitudinal fasciculus III (SLF III) (Figure 2(b)).

### 4.4. Predicting Motor Abilities and Recovery from Neuroanatomical Data

**Figure 3:**
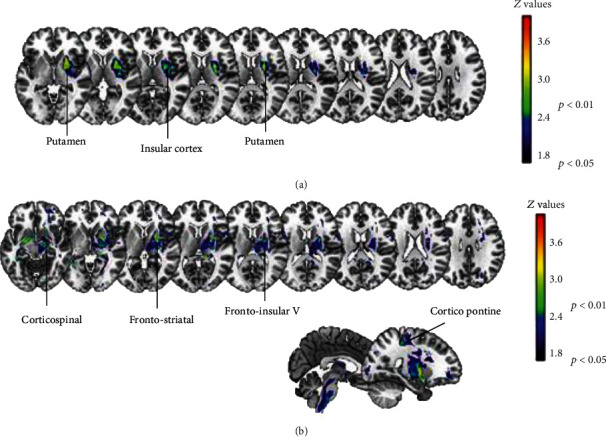
Significant brain-behavior associations observed between the pretreatment F-M UE scores and lesions (a) or white-matter disconnections (b).

In VLSM analysis, lower pretreatment motor performance was significantly associated with clusters of damaged voxels mainly located in putamen and insular cortex (Figure 3(a)), as well as to white matter disconnections within corticospinal tract, corticopontine, frontostriatal, and frontoinsular tract V (Figure 3(b), see Table 9S for detailed results in Supplementary materials).

**Figure 4:**
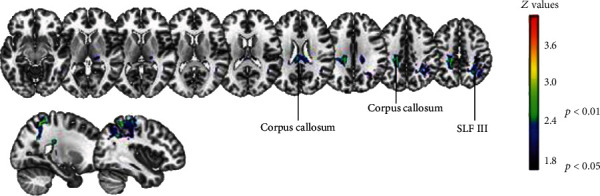
Significant brain-behavior associations observed between F-M UE recovery index and white-matter disconnections.

In the VLSM analysis for motor rehabilitation responsiveness, lower F-M UE recovery index was significantly associated with a wide parietal region. Even though significant results emerged in the lesion analysis for a small cluster located around the central gyrus, they were present in less than 50% of patients. In contrast, the white matter was found to be more reliably involved in motor outcome, especially across the SLFIII and the corpus callosum (Figure 4, see Table 10S for detailed results in Supplementary materials).

## 5. Discussion

In the neurological population, the rehabilitation of motor deficits relies on both sensorimotor and cognitive systems [2]. Voluntary motor behavior involves a wide neural network beyond motor [24, 28, 57] and attentional functions [32, 33]. However, the integrated investigation of motor, cognitive, and neuroanatomical factors that may influence motor recovery is still sparse.

The present retrospective study investigated whether attentional abilities influenced the outcome of motor rehabilitation, when controlling for clinical variables and for pretreatment sensorimotor skills. A second aim of the study was to assess which brain lesions and/or white-matter disconnections better predict the motor deficits and hinder the rehabilitation outcome. Even though sample size was small, all patients participated in TB rehabilitation programs for the upper limb in the context of clinical trials. This ensured consistency in the rehabilitation protocols and the availability of a detailed assessment of motor skills.

### 5.1. Sensorimotor System and Neuronal Correlates

In the linear regression model, for the whole sample of patients, the F-M UE recovery index was predicted by pretreatment sensorimotor abilities, attention, affected hemisphere, and age. This analysis highlights that some patients' characteristics contribute to interpatients' variability in responsiveness to motor rehabilitation. Notably, the model accounted for a large amount of the variability in the motor recovery index.

It is worth noting that upper limb sensorimotor residual abilities were summarized by the first principal component of a PCA conducted on all motor tests and scales. In line with previous studies that used the same approach [24, 71, 72], we observed that the first component accounts for a large amount of behavioral variance (here 76%). This is consistent with the idea that behavior is low-dimensional [71] and that a single “motor factor” adequately captures the residual motor abilities. Importantly, the motor factor influenced motor rehabilitation outcome. It is also worth noting that motor factor values are more influenced by pretreatment F-M UE and reaching performance scales than by simpler variables like sensation, proprioception, spasticity, and joints amplitude (see Table 4S in Supplementary materials for details). Considering that higher values of the motor factor index poorer motor performance and that the corresponding model regression weight was positive, it can be concluded that the performance gain (relative to pretreatment performance) yielded through rehabilitation was larger for patients with more severe upper limb motor difficulties. This suggests that patients with severe motor deficits have more “room for improvement” and it is consistent with the evidence that TB rehabilitations may boost upper limb motor amelioration [20] even for the most compromised patients.

The VLSM analyses related the patients' pretreatment F-M UE scores to lesions in sensory and motor areas, most notably the putamen and the insula. The putamen is considered as a primary motor structure, which is also necessary for higher-level motor processing, such as in mental rotation that relies on sensory memory and supports new learning [73]. The insula is a crucial area for cognitive processing of bodily awareness [74, 75] through the processing of various sensory internal stimuli [76, 77], but it is also involved in high-demanding attentional tasks and control, thanks to its interaction with large-scale brain networks [78].

In VLSM analyses on disconnection maps, the damage of the corticospinal tract and of some frontoparietal pathways (i.e., corticopontine, frontostriatal, and frontoinsular V tracts) emerged as predictors of the pretreatment motor abilities. The corticospinal tract is part of the main motor pathway, with a major role in controlling voluntary actions [79]. The involvement of other frontoparietal networks may instead suggest associations to other cognitive domains such as attention and language to monitor own motor execution and interact with external stimuli [28].

In relation to the lesion side, descriptive statistics revealed differences in the motor abilities between LBD and RBD patients, with the latter presenting higher severity of upper limb spasticity and reaching performance deficits. Lesion side influenced motor recovery outcome in the model. This might be related to small differences between LBD and RBD in the distribution of lesions affecting the primary sensorimotor systems.

Interestingly, the type of TB therapy did not enter into the model. This is in line with the fact that both TB methods are built on exercises of kinematic adaptation to continuous on-line feedback in ecological settings, as well as with the previous evidence that both methodologies boost upper limb motor recovery [17–20].

### 5.2. Cognitive System and Neuronal Correlates

Our regression modeling results show that selective attention skills (evaluated by the attentive matrices test) are positively related to the F-M UE recovery index. This result supports the hypothesis that preserved attention skills can positively impact the motor rehabilitation outcome, as motor and attention processes work together in motion [80].

Nevertheless, the complementary regression analysis carried out on the subgroup of patients with right hemisphere lesions suggests that the attentional modulation of the rehabilitation outcome is more specifically linked to visuospatial orienting as opposed to the more general selective attention. Further studies should exploit computerized assessment methods that can unveil more subtle visuospatial orienting deficits [81], even in LBD patients [37]. Spatial abilities are important for motor recovery of both RBD [33] and LBD patients [82], but unfortunately, our data did not include tests exploring other spatial processes such as apraxia [83].

The involvement of visuospatial attention is consistent with the results of the VLSM analysis on the motor recovery index (Figure 4). Indeed, SLF III is relevant in the intrahemispheric frontoparietal network supporting attentional orienting that has been associated with visuospatial neglect [71, 82]. Moreover, SLF III is thought to have a role in the link between the attention to salient stimuli and the planning of goal-directed actions [84]. The involvement of the corpus callosum seems instead to support the role of interhemispheric connectivity in stroke recovery, as previously reported for both motor deficits [57, 81] and visuospatial neglect [85, 86]. From the lesion analysis, a central parietal area was detected, but only in a small number of patients. This area may be linked to neglect severity [87].

Nevertheless, deficits in both motor and attention domains may stem from lesions inducing wide functional changes [72] in frontoparietal and interhemispheric connectivity [86]. Damage in SLF III and corpus callosum may support the idea of a widespread disruption of cortical activity in both motor and cognitive domains, disclosing the cooccurrence of attentional and motor impairments in stroke patients [33]. Indeed, the upper limb motor recovery of voluntary movements in our sample was supported by attention skills, which are also important for the higher-level cognitive processes of monitoring [6] and controlling [3] the motor execution.

In the same vein, SLF III was recently shown to be disconnected in stroke patients with anosognosia for hemiplegia [74], who overestimate their upper limb motor performance due to a lack of awareness for the motor impairment. Right hemisphere damage to the frontotemporal-parietal network disrupts the computational steps between motor planning and higher-level monitoring [88], affecting the level of awareness [89] and its fluctuations [90]. Even though anosognosia and neglect are mainly investigated in RBD patients, they can also occur following left hemisphere damage [37, 91] and are well known to negatively impact motor and cognitive recovery [92, 93].

Finally, patients' age was a significant predictor in the models, but its effect appears counterintuitive because it associated older age with higher values of the F-M UE recovery index. It should be noted, however, that the mean age was relatively high (62.414 ± 11.879 years) and the results might have been influenced by other demographic or clinical variables (note also that there was no correlation between age and pretreatment motor deficit; see full correlation matrix in Table 5S of the supplementary materials).

### 5.3. Study Limitation

The main limitation of the study is the relatively small sample size. This is due to the retrospective design and to the fact that patients underwent tailored assessments. This prevented the inspection of a broader range of cognitive domains. Moreover, we only considered variables without missing data in order to examine effects for the whole group and overcome model convergence issues. Similarly, for neuroanatomical analysis, we applied univariate statistical methods as suggested for lesion investigations in small samples, thereby ensuring a high specification in resulting clusters [66]. Despite univariate and multivariate brain-behavior mapping approaches have been shown to produce highly similar results [24], a bigger sample and the use of multivariate machine learning methods would have strengthened the generalization of our findings. Additionally, the severity of disconnections could be estimated more directly using other methods (e.g., [94]). Future studies should exploit a prospective design to collect information on a broader range of sensorimotor and cognitive skills, as well as multimodal neuroimaging data, to predict motor recovery in a large sample of patients.

## 6. Conclusion

The present retrospective study aimed to integrate clinical, behavioral, and neuroimaging data as predictors of upper limb motor recovery, exploiting a relatively small but selected sample of patients that consistently received TB motor rehabilitation. Results showed that age, hemisphere, pretreatment motor, and attentional abilities are associated with motor rehabilitation outcome. The integration of motor and cognitive variables is crucial to understand patients' variability in rehabilitation. For example, attention deficits, in particular visuospatial orienting, could play a key role into motor recovery of the upper limb, supporting rehabilitation's engagement and final outcome.

Brain-behavior mapping showed that frontoparietal areas are involved in both patients' residual motor abilities and recovery, but with different weighted contributions. While the pretreatment motor performance was more connected to motor areas and pathways, motor rehabilitation outcome was predicted from both motor and attentional networks.

In conclusion, the integration of behavioral and neuroanatomical information is a valuable approach to understand and tailor upper limb motor treatment in stroke patients. The possibility of predicting rehabilitation outcomes might inform clinical decisions on the intervention program, thereby optimizing resources and fostering patients' recovery.

## Figures and Tables

**Table 1:** Values for whole sample and divided for damaged hemisphere.

Test	Total	LBD	RBD	LBD vs. RBD comparison
Sensorimotor abilities				
Pretreatment F-M UE	32.07 ± 16.16	41.73 ± 16.24	26.17 ± 13.32	*p* = 0.015∗
Posttreatment F-M UE	38.52 ± 17.52	48.82 ± 16.39	32.22 ± 15.39	*p* = 0.013∗
F-M UE recovery index	24.01 ± 21.44	20.60 ± 12.68	26.10 ± 25.50	*p* = 0.446
Modified Ashworth	3.55 ± 3.62	1.09 ± 2.21	5.05 ± 3.52	*p* = 0.002∗
Reaching performance	17 ± 12.73	24.45 ± 11.85	12.44 ± 11.24	*p* = 0.011∗
Sensation	18.48 ± 6.43	20.82 ± 4.31	17.05 ± 7.18	*p* = 0.220
Joint amplitude	40.83 ± 6.08	42.64 ± 5.70	39.72 ± 6.20	*p* = 0.182
Type of TB (% virtual reality)	79.31%	81.82%	77.78%	*p* = 0.238
Attentional abilities				
Attentional matrices	38.31 ± 12.86	39.09 ± 11.48	37.83 ± 13.94	*p* = 0.794
BIT Stars			45.64 ± 14.11	

Note: Patients with LBD: left brain damage; RBD: right brain damage; F-M UE: Fugl-Meyer Upper-Extremity Fugl-Meyer test; *p*: *p* value; ^∗^: significant result.

**Table 2:** Significant regression model.

Independent variables	Est. Coeff.	St. Coeff.	Std. Err.	*t* value	*p* value
Intercept	-0.382	-0.382	-0.199	-1.992	0.0.058′
Age	0.013	0.722	2.609e^−3^	4.997	<0.0001^∗∗∗^
Lesion volume	-3.915e^−6^	-0.240	-2.048e^−6^	-1.912	0.068′
Affected hemisphere	-0.198	-0.457	-0.069	-2.858	0.009^∗∗^
Motor factor	0.142	0.662	0.031	4.491	0.0002^∗∗∗^
Attention	4.487e^−3^	0.269	2.143e^−3^	2.094	0.047^∗^

Note: Est. Coeff.: estimated coefficient; St. Coeff.: standardized coefficient; Std. Err.: standard error. Affected hemisphere is coded as 1 = left and 2 = right. *p* values: ^∗∗∗^ <0.001, ^∗∗^ <0.01, ^∗^ <0.05, ′ <0.10.

**Table 3:** Significant regression model for the subgroup of right brain damage patients.

Independent variables	Est. Coeff.	St. Coeff.	Std. Err.	*t* value	*p* value
Intercept	-1.417	-1.417	0.318	-4.462	0.0008^∗∗∗^
Age	0.018	0.891	0.003	4.973	0.0003^∗∗∗^
Time from onset	0.022	0.429	0.009	2.389	0.034^∗^
BIT Stars	0.007	0.374	0.003	2.412	0.033^∗^

Note: Est. Coeff.: estimated coefficient; St. Coeff.: standardized coefficient; Std. Err.: standard error; *p* values: ^∗∗∗^ <0.001, ^∗^ <0.05.

## Data Availability

The data that support the findings of this study are available from the corresponding author upon reasonable request.

## Supplementary Materials

Supplementary Materials In supplementary materials details of patients' demographic, clinical and experimental information (Table 1S-3S). Details of PCA (Figure 1S, Table 4S), correlation matrix (Table 5S, 6S), regression (Table 7S, 8S), and VLSM analyses (Table 8S-11S Figure 2S).
