# Supplementary material for: Sensorimotor, Attentional, and Neuroanatomical Predictors of Upper Limb Motor Deficits and Rehabilitation Outcome after Stroke
Source: Neural Plast. 2021 Apr 1;2021:8845685. doi: 10.1155/2021/8845685 (PMC8035034; doi:10.1155/2021/8845685)
Supplement: Supplementary Materials — In supplementary materials details of patients' demographic, clinical and experimental information (Table 1S-3S). Details of PCA (Figure 1S, Table 4S), correlation matrix (Table 5S, 6S), regression (Table 7S, 8S), and VLSM analyses (Table 8S-11S Figure 2S). [file 8845685.f1.zip › TABLE 7S.docx]

**Best subset regression**

Best subset regression was used as an alternative method to assess the robustness of the stepwise regression modelling results, in which variable selection and parameter estimation is known to be potentially biased. Though a best subset selection, all possible models were generated by assembling different number of predictors (from 1 to 10 available variables) and their goodness-of-fit was assessed using adjusted R^2^, Mallow’s C_P_ (C_P_) and Bayesian Information Criterion (BIC). The analysis was run with R using the function regsubsets from the package leaps [95]. The number of predictors in the best fitting model varied according to the goodness-of-fit measure. The model included 9 predictors using adjusted R^2^, 8 predictors using C_P_ and 5 predictors using BIC (Table 7S). Notably, the most conservative model (using BIC for model selection) had the same 5 predictors obtained by the step-wise analysis reported in the main text.

| TABLE 7S. Combination of variables from best subset regression | | | | | | | |  |
| --- | --- | --- | --- | --- | --- | --- | --- | --- |
| **Independent variables** | **BIC** | | **C_P_** | | | **R^2^** | | |
|  | **Std. Coeff.** | **p-value** | **Std. Coeff.** | **p-value** | **Std. Coeff.** | | **p-value** | |
| Intercept | -0.382 | 0.058’ | -0.540 | 0.21* | -0.419 | | 0.091’ | |
| Age | 0.722 | <0.001*** | 0.883 | <0.001*** | 0.896 | | <0.001*** | |
| Lesion volume | -0.240 | 0.068’ | -0.316 | 0.018* | -0.290 | | 0.029* | |
| Affected Hemisphere | -0.457 | 0.009** | -0.585 | 0.001* | -0.581 | | 0.001* | |
| Motor factor | 0.662 | 0.0002*** | 0.656 | <0.001*** | 0.613 | | 0.003** | |
| Attention | 0.269 | 0.047* | 0.299 | 0.022* | 0.288 | | 0.026* | |
| Time from onset | - | - | 0.249 | 0.100 | 0.277 | | 0.070’ | |
| Gender | - | - | -0.217 | 0.093’ | -0.216 | | 0.092’ | |
| Education | - | - | 0.197 | 0.113 | 0.178 | | 0.148 | |
| Type of RHB | - | - | - | - | -0.145 | | 0.231 | |

Note: Std. Coeff.= Standardized Coefficient; p-values are coded as: ***<0.001, **<0.01, *<0.05, ’<0.10.
