# Supplementary material for: Sensorimotor, Attentional, and Neuroanatomical Predictors of Upper Limb Motor Deficits and Rehabilitation Outcome after Stroke
Source: Neural Plast. 2021 Apr 1;2021:8845685. doi: 10.1155/2021/8845685 (PMC8035034; doi:10.1155/2021/8845685)
Supplement: Supplementary Materials — In supplementary materials details of patients' demographic, clinical and experimental information (Table 1S-3S). Details of PCA (Figure 1S, Table 4S), correlation matrix (Table 5S, 6S), regression (Table 7S, 8S), and VLSM analyses (Table 8S-11S Figure 2S). [file 8845685.f1.zip › TABLE 11S.docx]

| TABLE 11S. Associations to post-treatment F-M UE. | | | | |
| --- | --- | --- | --- | --- |
| **Areas** | **Damaged voxel** | **% Damage** | **N** | **Z** |
| Putamen | 4313 | 50.58 | 16 | 3.89 |
| Insula cortex | 1475 | 4.70 | 16 | 3.54 |
| **Tracts** |  |  |  |  |
| Corticospinal | 21049 | 20.29 | 26 | 3.61 |
| Corticopontine | 20721 | 17.60 | 25 | 3,61 |
| Fronto striatal | 22778 | 11.55 | 25 | 3,61 |
| Fronto insular tract V | 1955 | 10.49 | 26 | 3,61 |
| Superior longitudinal III | 9446 | 4.81 | 25 | 3,61 |
| Corpus callosum | 32966 | 2.74 | 27 | 3,62 |

Note: Damaged voxel = number of damaged voxels, % Damage = percentage of area with damage, N = Number of patients with damage in the cluster, Z= peak of Z value.
