# Supplementary material for: Sensorimotor, Attentional, and Neuroanatomical Predictors of Upper Limb Motor Deficits and Rehabilitation Outcome after Stroke
Source: Neural Plast. 2021 Apr 1;2021:8845685. doi: 10.1155/2021/8845685 (PMC8035034; doi:10.1155/2021/8845685)
Supplement: Supplementary Materials — In supplementary materials details of patients' demographic, clinical and experimental information (Table 1S-3S). Details of PCA (Figure 1S, Table 4S), correlation matrix (Table 5S, 6S), regression (Table 7S, 8S), and VLSM analyses (Table 8S-11S Figure 2S). [file 8845685.f1.zip › TABLE 10S.docx]

| TABLE 10S. Associations to F-M UE recovery index. | | | |  | |  |
| --- | --- | --- | --- | --- | --- | --- |
| **Areas** | **Damaged voxel** | **% Damage** | **N** | | **Z** | |
| Precentral gyrus | 2825 | 2.61 | 8 | | 3.06 | |
| Postcentral gyrus | 2072 | 2.54 | 9 | | 3.06 | |
| **Tracts** |  |  |  | |  | |
| Superior longitudinal III | 17459 | 8.88 | 25 | | 3.61 | |
| Corpus callosum | 48018 | 4.08 | 27 | | 3,61 | |
| Cortico spinal | 2799 | 2.69 | 26 | | 3,61 | |
| Cortico-pontine | 2018 | 1.71 | 25 | | 3.61 | |
| Fronto striatal | 2619 | 1.33 | 25 | | 3.61 | |

Note: Damaged voxel = number of damaged voxels, % Damage = percentage of area with damage, N = Number of patients with damage in the cluster, Z= peak of Z value.
