# Supplementary material for: Sensorimotor, Attentional, and Neuroanatomical Predictors of Upper Limb Motor Deficits and Rehabilitation Outcome after Stroke
Source: Neural Plast. 2021 Apr 1;2021:8845685. doi: 10.1155/2021/8845685 (PMC8035034; doi:10.1155/2021/8845685)
Supplement: Supplementary Materials — In supplementary materials details of patients' demographic, clinical and experimental information (Table 1S-3S). Details of PCA (Figure 1S, Table 4S), correlation matrix (Table 5S, 6S), regression (Table 7S, 8S), and VLSM analyses (Table 8S-11S Figure 2S). [file 8845685.f1.zip › TABLE 9S.docx]

**Neuroanatomical results from the VLSM analyses**

Detailed information about VLSM results are reported in Tables 9S and 10S. For descriptive purpose, we included only the most frequently lesioned regions and tracts across patients (i.e., in more than 50% and 80% respectively) and for each region/tract we report the percentage of damage.

| TABLE 9S. Associations to pre-treatment F-M UE. | | | | |
| --- | --- | --- | --- | --- |
| **Areas** | **Damaged voxel** | **% Damage** | **N** | **Z** |
| Putamen | 6244 | 73.37 | 16 | 3.89 |
| Insula cortex | 2601 | 8.28 | 16 | 3.38 |
| **Tracts** |  |  |  |  |
| Corticospinal | 29075 | 27.80 | 26 | 3.61 |
| Cortico-pontine | 31709 | 26.59 | 25 | 3,61 |
| Fronto striatal | 35502 | 17.79 | 25 | 3,61 |
| Fronto insular tract V | 3104 | 16.76 | 26 | 3,61 |
| Superior longitudinal III | 15585 | 7.91 | 25 | 3,61 |
| Corpus callosum | 50081 | 4.17 | 27 | 3,62 |

Note: Damaged voxel = number of damaged voxels, % Damage = percentage of area with damage, N = Number of patients with damage in the cluster, Z = peak of Z value.
