# Supplementary material for: Sensorimotor, Attentional, and Neuroanatomical Predictors of Upper Limb Motor Deficits and Rehabilitation Outcome after Stroke
Source: Neural Plast. 2021 Apr 1;2021:8845685. doi: 10.1155/2021/8845685 (PMC8035034; doi:10.1155/2021/8845685)
Supplement: Supplementary Materials — In supplementary materials details of patients' demographic, clinical and experimental information (Table 1S-3S). Details of PCA (Figure 1S, Table 4S), correlation matrix (Table 5S, 6S), regression (Table 7S, 8S), and VLSM analyses (Table 8S-11S Figure 2S). [file 8845685.f1.zip › TABLE 8S.docx]

**Regression model on pre-treatment F-M UE scores**

A stepwise regression model was computed on the pre-treatment F-M scores to assess the influence of clinical and demographic variables on the initial motor performance. We entered demographic information (i.e., age, gender and education), clinical parameters (i.e., etiology, time from onset, affected hemisphere, lesion volume and type of TB motor training) and attentional deficits (values at the attentional matrices test) as predictors (Table 8S).

|  | TABLE 8S. Significant regression model on pre-treatment F-M UE. | | | | | |
| --- | --- | --- | --- | --- | --- | --- |
| **Independent variables** | | **Est. Coeff.** | **St.Coeff.** | **Std. Err.** | **t value** | **p-value** |
| Intercept | | 41.725 | 41.725 | 5.299 | 7.875 | <0.001*** |
| Time from onset | | -1.345 | -0.383 | 0.625 | -2.154 | 0.04* |

Note: Est. coeff.= Estimated Coefficient; St. Coeff. = Standardized Coefficient; Std. Err.= Standard Error. p-values coded as: ***<0.001, *<0.05.
