# Supplementary material for: Sensorimotor, Attentional, and Neuroanatomical Predictors of Upper Limb Motor Deficits and Rehabilitation Outcome after Stroke
Source: Neural Plast. 2021 Apr 1;2021:8845685. doi: 10.1155/2021/8845685 (PMC8035034; doi:10.1155/2021/8845685)
Supplement: Supplementary Materials — In supplementary materials details of patients' demographic, clinical and experimental information (Table 1S-3S). Details of PCA (Figure 1S, Table 4S), correlation matrix (Table 5S, 6S), regression (Table 7S, 8S), and VLSM analyses (Table 8S-11S Figure 2S). [file 8845685.f1.zip › TABLE 6S.docx]

| TABLE 6S. Correlation matrix within all variables for the subgroup of RBD patients | | | | | | | | | | | | |
| --- | --- | --- | --- | --- | --- | --- | --- | --- | --- | --- | --- | --- |
|  | F-M UE index | Age | Gender | Education | Etiology | Onset | Volume | TB | Attention | Motor factor | Pre-  F-M UE | BIT Stars |
| F-M UE index |  |  |  |  |  |  |  |  |  |  |  |  |
| Age | 0.57** |  |  |  |  |  |  |  |  |  |  |  |
| Gender | 0.01 | 0.09 |  |  |  |  |  |  |  |  |  |  |
| Education | -0.12 | 0.44 | 0.12 |  |  |  |  |  |  |  |  |  |
| Etiology | -0.03 | 0.06 | 0.23 | -0.11 |  |  |  |  |  |  |  |  |
| Onset | -0.04 | -0.50* | 0.31 | 0.15 | 0 |  |  |  |  |  |  |  |
| Volume | -0.40 | -0.35 | -0.07 | 0 | -0.22 | 0.22 |  |  |  |  |  |  |
| TB | -0.38 | -0.19 | 0.08 | -0.21 | -0.67*** | 0.24 | 0.09 |  |  |  |  |  |
| Attention | 0.06 | -0.19 | -0.09 | 0.44’ | -0.05 | -0.08 | -0.12 | -0.30 |  |  |  |  |
| Motor factor | -0.28 | -0.04 | -0.01 | 0.22 | 0.09 | -0.44’ | -0.19 | -0.06 | 0.50* |  |  |  |
| Pre- F-M UE | -0.22 | 0.05 | 0.06 | 0.10 | 0.08 | -0.51* | -0.22 | -0.16 | 0.40 | 0.92 |  |  |
| BIT Stars | 0.35 | 0 | -0.07 | 0.16 | 0.27 | -0.06 | -0.26 | -0.17 | 0.82*** | 0.23 | 0.29 |  |

Note: Cell values represent r for Pearson’s correlations and rho for Spearman’s rank correlations. Gender (1=Male, 2=Female), etiology (1=Ischemic, 2=Hemorrhagic), Onset = Time from onset (in months), hemisphere (1= LDB, 2=RBD), Volume = lesion volume, TB (Type: 1=Virtual reality software, 2=AMADEO robot) and pre- F-M UE = Pre-treatment F-M UE. Asterisks code p-values as follows:***<0.001, **<0.01, *<0.05, ‘<0.10
