# Supplementary material for: Sensorimotor, Attentional, and Neuroanatomical Predictors of Upper Limb Motor Deficits and Rehabilitation Outcome after Stroke
Source: Neural Plast. 2021 Apr 1;2021:8845685. doi: 10.1155/2021/8845685 (PMC8035034; doi:10.1155/2021/8845685)
Supplement: Supplementary Materials — In supplementary materials details of patients' demographic, clinical and experimental information (Table 1S-3S). Details of PCA (Figure 1S, Table 4S), correlation matrix (Table 5S, 6S), regression (Table 7S, 8S), and VLSM analyses (Table 8S-11S Figure 2S). [file 8845685.f1.zip › TABLE 5S.docx]

**Correlation matrix**

A correlation matrix within all collected variables is reported for the whole group (Table 5S) and for the subgroup of RDB patients (Table 6S). We computed Pearson’s correlations for continuous variables and Spearman’s rank correlations for ordinal variables (i.e., gender, etiology, affected hemisphere and type of TB).

| TABLE 5S. Correlation matrix within all variables for the whole sample of patients | | | | | | | | | | | | |
| --- | --- | --- | --- | --- | --- | --- | --- | --- | --- | --- | --- | --- |
|  | F-M UE index | Age | Gender | Edu-cation | Etio-logy | Onset | Hemi-sphere | Volu-me | TB | Atten-tion | Motor factor | Pre-  F-M UE |
| F-M UE index |  |  |  |  |  |  |  |  |  |  |  |  |
| Age | 0.52** |  |  |  |  |  |  |  |  |  |  |  |
| Gender | 0.04 | -0.01 |  |  |  |  |  |  |  |  |  |  |
| Education | 0.04 | -0.13 | 0.12 |  |  |  |  |  |  |  |  |  |
| Etiology | 0.01 | -0.12 | 0.49** | -0.13 |  |  |  |  |  |  |  |  |
| Onset | -0.05 | -0.33’ | 0.18 | 0.22 | -0.04 |  |  |  |  |  |  |  |
| Hemi-sphere | 0.10 | 0.36’ | 0.06 | 0.10 | -0.06 | 0.21 |  |  |  |  |  |  |
| Volume | -0.33՚ | -0.15 | -0.28 | -0.02 | -0.23 | 0.23 | -0.06 |  |  |  |  |  |
| TB | -0.33’ | 0.01 | -0.03 | -0.17 | -0.51** | 0.03 | -0.05 | 0.01 |  |  |  |  |
| Attention | 0.06 | -0.26 | -0.02 | 0.07 | 0.08 | -0.10 | -0.03 | -0.08 | -0.10 |  |  |  |
| Motor factor | 0.38* | 0 | 0.02 | 0.01 | 0 | 0.37* | 0.50** | 0.11 | -0.18 | -0.10 |  |  |
| Pre- F-M UE | -0.37* | 0.02 | 0.03 | -0.06 | 0.08 | -0.38* | -0.48** | -0.14 | 0.13 | 0.07 | -0.97*** |  |

Note: Cell values represent r for Pearson’s correlations and rho for Spearman’s rank correlations. Gender (1=Male, 2=Female), etiology (1=Ischemic, 2=Hemorrhagic), Onset = Time from onset (in months), hemisphere (1= LDB, 2=RBD), Volume = lesion volume, TB (Type: 1=Virtual reality software, 2=AMADEO robot) and pre- F-M UE = Pre-treatment F-M UE. Asterisks code p-values as follows: ***<0.001, **<0.01, *<0.05, ‘<0.10
