# Supplementary material for: Sensorimotor, Attentional, and Neuroanatomical Predictors of Upper Limb Motor Deficits and Rehabilitation Outcome after Stroke
Source: Neural Plast. 2021 Apr 1;2021:8845685. doi: 10.1155/2021/8845685 (PMC8035034; doi:10.1155/2021/8845685)
Supplement: Supplementary Materials — In supplementary materials details of patients' demographic, clinical and experimental information (Table 1S-3S). Details of PCA (Figure 1S, Table 4S), correlation matrix (Table 5S, 6S), regression (Table 7S, 8S), and VLSM analyses (Table 8S-11S Figure 2S). [file 8845685.f1.zip › TABLE 4S.docx]

Table 4S shows how the different sensorimotor tests loaded on the first principal component. Higher loading is found for F-M UE and reaching performance scales, in which lower values mean higher performance. Note that increasing rank in the sensorimotor scales index improving performance, with the exception of the modified Ashworth scale that goes in the opposite direction.

| TABLE 4S. Loading on the first principal component |  |
| --- | --- |
| **Sensorimotor assessment** | **1° component** |
| Modified Ashworth scale | 0.105 |
| Reaching Performance scale | -0.578 |
| Sensation | -0.122 |
| Joint amplitude | -0.126 |
| Pre-treatment F-M UE | -0.790 |
