# Supplementary material for: Sensorimotor, Attentional, and Neuroanatomical Predictors of Upper Limb Motor Deficits and Rehabilitation Outcome after Stroke
Source: Neural Plast. 2021 Apr 1;2021:8845685. doi: 10.1155/2021/8845685 (PMC8035034; doi:10.1155/2021/8845685)
Supplement: Supplementary Materials — In supplementary materials details of patients' demographic, clinical and experimental information (Table 1S-3S). Details of PCA (Figure 1S, Table 4S), correlation matrix (Table 5S, 6S), regression (Table 7S, 8S), and VLSM analyses (Table 8S-11S Figure 2S). [file 8845685.f1.zip › FIGURE 2S.docx]

**Additional VLSM analyses with post-treatment F-M UE**

VLSM analyses were computed on post-treatment F-M UE, with pre-treatment F-M UE and lesion volume as covariates (Figure 2S). Detailed information about significant damage on lesion and disconnection maps are reported in Tables 11S. The resulting maps show high similarity with those for the pre-treatment F-M UE. This finding is likely to reflect the fact the scores do not directly measure patients’ recovery.


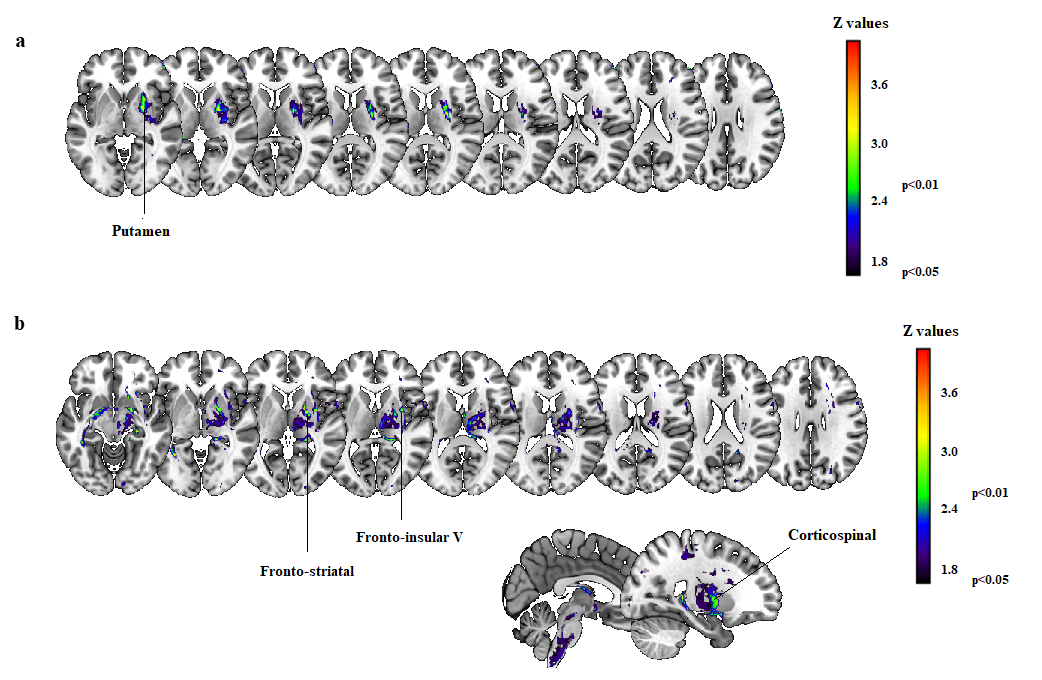


FIGURE 2S. Significant brain-behavior associations observed between the post-treatment F-M UE scores and lesions (a) or white-matter disconnections (b).
