# Supplementary material for: Sensorimotor, Attentional, and Neuroanatomical Predictors of Upper Limb Motor Deficits and Rehabilitation Outcome after Stroke
Source: Neural Plast. 2021 Apr 1;2021:8845685. doi: 10.1155/2021/8845685 (PMC8035034; doi:10.1155/2021/8845685)
Supplement: Supplementary Materials — In supplementary materials details of patients' demographic, clinical and experimental information (Table 1S-3S). Details of PCA (Figure 1S, Table 4S), correlation matrix (Table 5S, 6S), regression (Table 7S, 8S), and VLSM analyses (Table 8S-11S Figure 2S). [file 8845685.f1.zip › FIGURE 1S.docx]

**PCA**

Principal component analysis (PCA) was run on all available pre-treatment motor assessment scores. In line with previous studies, we used the first principal component as “motor factor” because it accounted for 75.9% of the variance [24] (Figure 1S). That is, the PCA strongly reduced the dimensionality of the data so that the first component preserved adequate information on the residual motor abilities. PCA scores were subsequently normalized to get a breaking “0” value between generally spared and impaired abilities [57].


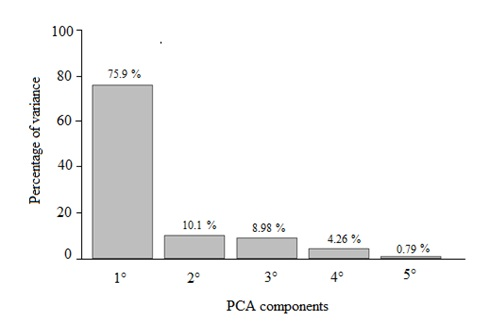


FIGURE 1S. Principal Component Analysis (PCA). The bar plot shows the percentage of variance explained by each component.
