# Supplementary material for: Sensorimotor, Attentional, and Neuroanatomical Predictors of Upper Limb Motor Deficits and Rehabilitation Outcome after Stroke
Source: Neural Plast. 2021 Apr 1;2021:8845685. doi: 10.1155/2021/8845685 (PMC8035034; doi:10.1155/2021/8845685)
Supplement: Supplementary Materials — In supplementary materials details of patients' demographic, clinical and experimental information (Table 1S-3S). Details of PCA (Figure 1S, Table 4S), correlation matrix (Table 5S, 6S), regression (Table 7S, 8S), and VLSM analyses (Table 8S-11S Figure 2S). [file 8845685.f1.zip › TABLE 3S.docx]

| TABLE 3S. Values for whole sample and divided for damaged hemisphere. | | | | |
| --- | --- | --- | --- | --- |
| **Test** | **Total** | **LBD**  **(n = 11)** | **RBD**  **(n = 18)** | **LBD vs. RBD**  **comparison** |
| General cognitive state | 26.95±1.75 | 28±1 (5) | 26.7±1.81 (16) | p=0.149 |
| Reasoning | 26.34±6.27 | 29.09±4.16 (11) | 24.33±6.89 (15) | p=0.039* |
| Memory - Short term | 5.78±1.13 | 5.6±0.89 (5) | 5.83±1.20 (18) | P=0.646 |
| Memory - Long term | 14.67±7.31 | 18.68±7.29 (7) | 11.55±5.95 (9) | p=0.059 |
| Working Memory | 4.09±1.24 | 3.8±1.30 (5) | 4.17±1.25 (18) | p=0.700 |
| Constructional apraxia-simple | 9.5±3.15 | 10.43±1.90 (7) | 8.91±3.70 (11) | p=0.270 |
| Constructional apraxia-complex | 27.32±8.48 | 31.62±6.02 (8) | 23.5±8.78 (9) | p=0.037* |

Note: Number of patients for each test is shown in parentheses; LBD = Left and RBD = Right damaged patients; p = p-value, *= significant result for p<.05.
