# Supplementary material for: Sensorimotor, Attentional, and Neuroanatomical Predictors of Upper Limb Motor Deficits and Rehabilitation Outcome after Stroke
Source: Neural Plast. 2021 Apr 1;2021:8845685. doi: 10.1155/2021/8845685 (PMC8035034; doi:10.1155/2021/8845685)
Supplement: Supplementary Materials — In supplementary materials details of patients' demographic, clinical and experimental information (Table 1S-3S). Details of PCA (Figure 1S, Table 4S), correlation matrix (Table 5S, 6S), regression (Table 7S, 8S), and VLSM analyses (Table 8S-11S Figure 2S). [file 8845685.f1.zip › TABLE 2S.docx]

| TABLE 2S. Experimental data for each patient. | | | | | | | | | | |
| --- | --- | --- | --- | --- | --- | --- | --- | --- | --- | --- |
| **Patient** | **Attentional**  **matrices**  **(cut-off >30)** |  | **BIT**  **(cut-off >129)** |  | **BIT -**  **Star cancellation**  **(cut-off >50)** |  | **Type**  **of TB** | **pre-treatment**  **F-M UE** | **post-treatment**  **F-M UE** | **F-M UE**  **index** |
| 1 | 32 |  | **83** |  | n.a. |  | 1 | 21 | 33 | 57.14 |
| 2 | 50 |  | 136 |  | *50* |  | 2 | 9 | 13 | 44.44 |
| 3 | 33 |  | 145 |  | 54 |  | 1 | 55 | 61 | 10.91 |
| 4 | 50 |  | n.a. |  | n.a. |  | 1 | 55 | 63 | 14.55 |
| 5 | 38 |  | 145 |  | 54 |  | 2 | 17 | 24 | 41.18 |
| 6 | 37 |  | **127** |  | **43** |  | 1 | 23 | 33 | 43.48 |
| 7 | 43 |  | 146 |  | 54 |  | 1 | 25 | 32 | 28 |
| 8 | 56 |  | 133 |  | 54 |  | 2 | 42 | 55 | 30.35 |
| 9 | 47 |  | 140 |  | 54 |  | 2 | 48 | 58 | 20.83 |
| 10 | 48 |  | 146 |  | 54 |  | 1 | 33 | 33 | 0 |
| 11 | 35 |  | 137 |  | *51* |  | 1 | 15 | 27 | 0.8 |
| 12 | **15** |  | **128** |  | n.a. |  | 1 | 51 | 60 | 17.65 |
| 13 | 34 |  | **125** |  | *50* |  | 2 | 17 | 27 | 58.82 |
| 14 | 36 |  | 145 |  | 54 |  | 1 | 31 | 43 | 38.71 |
| 15 | 51 |  | n.a. |  | n.a. |  | 1 | 20 | 26 | 30 |
| 16 | **8** |  | **33** |  | **8** |  | 1 | 26 | 27 | 3 |
| 17 | 48 |  | 138 |  | 52 |  | 1 | 24 | 32 | 33.33 |
| 18 | **26** |  | **86** |  | **17** |  | 1 | 13 | 13 | 0 |
| 19 | 41 |  | 131 |  | 52 |  | 1 | 7 | 7 | 0 |
| 20 | 56 |  | 144 |  | 54 |  | 1 | 40 | 41 | 2.5 |
| 21 | 42 |  | n.a. |  | n.a. |  | 1 | 53 | 53 | 0 |
| 22 | **21** |  | n.a. |  | n.a. |  | 1 | 60 | 66 | 10 |
| 23 | **28** |  | **86** |  | 52 |  | 1 | 31 | 44 | 41.94 |
| 24 | 36 |  | n.a. |  | n.a. |  | 1 | 53 | 64 | 20.75 |
| 25 | **23** |  | **104** |  | **33** |  | 1 | 16 | 15 | -6.3 |
| 26 | 58 |  | n.a. |  | n.a. |  | 1 | 55 | 65 | 18.18 |
| 27 | **22** |  | 142 |  | 54 |  | 2 | 35 | 40 | 14.29 |
| 28 | 53 |  | 136 |  | 54 |  | 1 | 38 | 38 | 0 |
| 29 | 44 |  | 134 |  | 52 |  | 1 | 17 | 24 | 41.18 |

Note: BIT = Behavioral Inattention Test; F-M UE = Fugl-meyer Upper-extremity; Bold = values under cut-off; *Italic* = values around cut-off; n.a.= not available; Type of TB: 1= Virtual reality software, 2= AMADEO robot.
