# Supplementary material for: Sensorimotor, Attentional, and Neuroanatomical Predictors of Upper Limb Motor Deficits and Rehabilitation Outcome after Stroke
Source: Neural Plast. 2021 Apr 1;2021:8845685. doi: 10.1155/2021/8845685 (PMC8035034; doi:10.1155/2021/8845685)
Supplement: Supplementary Materials — In supplementary materials details of patients' demographic, clinical and experimental information (Table 1S-3S). Details of PCA (Figure 1S, Table 4S), correlation matrix (Table 5S, 6S), regression (Table 7S, 8S), and VLSM analyses (Table 8S-11S Figure 2S). [file 8845685.f1.zip › TABLE 1S.docx]

**Patients’ information**

Patients’ demographic and clinical information is reported in Table 1S. Behavioral data on motor performance index and attention tests are reported in Table 2S. Other neuropsychological tests were not available for the whole sample, but for descriptive purpose we reported in Table 3S those that were relatively common across patients.

| TABLE 1S. Demographic and clinical information for each patient. | | | | | | | | | | |
| --- | --- | --- | --- | --- | --- | --- | --- | --- | --- | --- |
| **Patient** | **Age** | **Gender** | **Education** |  | **Affected**  **hemisphere** |  | **Etiology** |  | **Lesion size**  **(mm^3^)** | **Time from onset**  **(months)** |
| 1 | 76 | M | 18 |  | RH |  | I |  | 2505 | 3.07 |
| 2 | 66 | M | 17 |  | RH |  | I |  | 4142 | 5.80 |
| 3 | 64 | M | 17 |  | LH |  | I |  | 29822 | 10.73 |
| 4 | 59 | M | 13 |  | LH |  | I |  | 2484 | 3.60 |
| 5 | 41 | M | 13 |  | LH |  | I |  | 3815 | 10.63 |
| 6 | 81 | F | 12 |  | RH |  | I |  | 16967 | 3.30 |
| 7 | 49 | F | 13 |  | LH |  | H |  | 445 | 9.77 |
| 8 | 72 | M | 13 |  | RH |  | H |  | 665 | 2.50 |
| 9 | 63 | F | 13 |  | RH |  | H |  | 15637 | 8.03 |
| 10 | 57 | M | 13 |  | RH |  | I |  | 236 | 1.97 |
| 11 | 79 | M | 5 |  | RH |  | I |  | 248 | 12.63 |
| 12 | 79 | F | 18 |  | RH |  | I |  | 204 | 9.20 |
| 13 | 73 | M | 5 |  | RH |  | H |  | 5892 | 6.70 |
| 14 | 62 | M | 13 |  | LH |  | I |  | 10655 | 1.30 |
| 15 | 61 | M | 8 |  | LH |  | I |  | 17646 | 3.17 |
| 16 | 67 | M | 8 |  | RH |  | I |  | 3237 | 5.80 |
| 17 | 82 | F | 8 |  | RH |  | I |  | 1792 | 9.43 |
| 18 | 59 | M | 13 |  | RH |  | I |  | 53970 | 11.23 |
| 19 | 44 | M | 8 |  | RH |  | I |  | 42779 | 15.67 |
| 20 | 53 | M | 12 |  | RH |  | I |  | 13979 | 10.50 |
| 21 | 47 | M | 8 |  | LH |  | I |  | 283 | 6.13 |
| 22 | 65 | M | 5 |  | LH |  | I |  | 13360 | 2.07 |
| 23 | 81 | M | 8 |  | RH |  | I |  | 16725 | 1.80 |
| 24 | 66 | M | 8 |  | LH |  | I |  | 17141 | 9.30 |
| 25 | 56 | F | 8 |  | RH |  | I |  | 1729 | 13.13 |
| 26 | 57 | F | 5 |  | LH |  | H |  | 1926 | 1.57 |
| 27 | 53 | F | 13 |  | LH |  | H |  | 6360 | 5.23 |
| 28 | 50 | M | 13 |  | RH |  | I |  | 20198 | 5 |
| 29 | 48 | F | 23 |  | RH |  | I |  | 3376 | 18.90 |

Note: M= male, F= female, RH= right hemisphere, LH=left hemisphere, I= ischemic, H= hemorrhagic.
